# Screen Time as an Indirect Factor in Childhood Obesity: A Narrative Review

**DOI:** 10.3390/nu18142261

**Published:** 2026-07-10

**Authors:** Patrycja Giefert, Weronika Wojton, Katarzyna Dereń

**Affiliations:** Faculty of Health Sciences and Psychology, Collegium Medicum, University of Rzeszów, 35-959 Rzeszów, Poland; wwojton@ur.edu.pl (W.W.); kderen@ur.edu.pl (K.D.)

**Keywords:** screen time, digital media, obesity, overweight, children, adolescents

## Abstract

**Background/Objectives**: Despite existing guidelines on limiting screen time, children and adolescents are spending an increasing number of hours in front of digital devices. This increase raises concerns about long-term health consequences, particularly in the context of the growing prevalence in childhood overweight and obesity. The aim of this review is to present the current state of knowledge regarding the relationship between increased screen time and weight-related health outcomes in the pediatric population. It also highlights the need for health education targeting both children and parents, based primarily on developing skills to manage time spent in front of electronic screens. **Methods**: A narrative review of the literature was conducted in the MEDLINE (PubMed), Web of Science and Scopus databases, including studies published between 2015 and 2025. **Results**: Available evidence suggests that prolonged screen exposure may be associated with reduced physical activity, circadian rhythm disturbances, including sleep problems, increased stress levels, and adverse mental health outcomes. These factors may interact and reinforce one another, potentially contributing to a positive energy balance and an increased risk of overweight and obesity among young people. **Conclusions**: The evidence reviewed highlights the ongoing digitalisation of younger generations and the potential consequences of excessive screen use. The underlying mechanisms appear complex and multidirectional. However, methodological heterogeneity across studies underscores the need for well-designed longitudinal and intervention research.

## 1. Introduction

Over the past five decades, the prevalence of overweight and obesity among children and adolescents has increased steadily [1,2], and this phenomenon is now widely recognized as a global epidemic. According to recent data from the World Health Organization (WHO), in 2022 more than 390 million individuals aged 5–19 years were overweight, including 160 million living with obesity [3]. Other reports indicate that over the past 40 years, the number of children and adolescents with obesity has increased more than tenfold [4]. This marks a substantial rise compared with 1990, when 31 million individuals in this age group were affected by obesity [3]. Importantly, projections remain concerning. Estimates from the World Obesity Federation (2019) suggest that the number of children aged 5–19 years living with obesity may reach 254 million by 2030 [5]. Whilst weight disorders may have multiple underlying causes, including genetic, behavioural and environmental factors [1], research suggests that excessive screen time is a significant, modifiable risk factor for overweight and obesity among children and adolescents [6].

With the rapid advancement of technology, contemporary children and adolescents are growing up in an environment characterized by widespread and virtually unrestricted access to electronic devices, with screens becoming an integral part of daily life [7,8]. Consequently, the amount of time spent on screen-based activities continues to increase. A major factor contributing to this trend was the COVID-19 pandemic, during which social isolation resulted in the migration of many aspects of everyday life to digital environments [9,10]. The use of screen-based devices is often necessary for educational purposes, and moderate recreational use may have beneficial effects on the well-being of young people [11]. However, over recent decades, the time devoted to such activities has increased substantially, and the majority of children now exceed recommended guidelines [11,12]. Current evidence indicates that adolescents aged 12–17 years spend at least 4 h per day engaged in screen-based activities [13], while among younger children, this duration typically ranges from 2 to 3 h per day [8]. These trends raise significant concerns regarding the long-term implications for the health and well-being of the younger population, including an increased risk of chronic diseases, as well as potential adverse effects on emotional and mental health.

The relationship between prolonged use of screen-based devices and childhood overweight and obesity is complex. Although screen exposure does not directly cause weight gain, it may act as a mediating factor influencing children’s behaviours and thereby increasing the risk of overweight and obesity. To date, the underlying mechanisms have not been fully elucidated, and findings across studies remain inconsistent. Nevertheless, the available evidence indicates a positive association between increased screen time and weight-related outcomes among children and adolescents [14,15,16,17]. A growing body of research has linked excessive screen time with a range of adverse health effects, including sleep disturbances, fatigue, and symptoms of stress and anxiety, which may in turn contribute to weight gain in the pediatric population [18,19]. Importantly, frequent use of devices such as smartphones, tablets, computers, and televisions is often associated with a more sedentary lifestyle. This, in turn, reduces the time allocated to physical activity, leading to lower energy expenditure, which directly contributes to weight gain [20].

Although the association between screen time and childhood overweight and obesity has been the subject of numerous studies, the mediating mechanisms underlying this relationship remain incompletely understood. To date, the roles of sleep disturbances, mental health problems, and reduced physical activity have most often been examined separately. Therefore, this narrative review aims to provide a comprehensive synthesis of these interconnected mechanisms and their potential contribution to the development of overweight and obesity in children and adolescents. A better understanding of these relationships may support the development of effective prevention strategies and contribute to creating a healthier digital environment for children and adolescents.

## 2. Methodological Approach

This article is a narrative review. This approach was chosen due to the broad and multidimensional nature of the topic, which makes a systematic review focused on a single, narrowly defined research question difficult to conduct. Consequently, PRISMA-based selection procedures and quantitative reporting of records at individual stages of the selection process were not applied. The aim of the review was to synthesize and contextualize current evidence regarding screen time, including both recreational and educational screen use, as a potential mediating factor in childhood obesity, with particular emphasis on pathways involving sleep disturbances, mental health outcomes, and physical activity.

The collected evidence was critically evaluated and synthesized. To enhance the reliability of the review and minimize potential bias, two authors independently assessed the relevance of the identified publications in relation to the scope and objectives of the review. Any disagreements were resolved through discussion and, when necessary, consultation with a third author.

Between 27 February and 29 March 2026, a comprehensive literature search was conducted in MEDLINE (PubMed), Web of Science, Scopus, ScienceDirect, and Google Scholar. The search strategy included combinations of the following keywords: “screen time”, “digital media”, “screens”, “duration of screen use”, “social media”, “overweight”, “obesity”, “childhood obesity”, “sleep deprivation”, “sleep disorders”, “depression”, “stress”, “anxiety”, “mental disorders”, “emotional disorders”, and “physical activity”.

Eligibility criteria were established before the literature screening process and were based on the objectives of the review. Publications were included if they: (1) were written in English or Polish; (2) examined screen time as a potential factor associated with overweight and obesity in children and adolescents, either directly or indirectly; and (3) represented various types of evidence, including systematic reviews, meta-analyses, narrative reviews, scoping reviews, observational studies, and clinical trials published between 2015 and 2025. Selected publications published before 2015 were also included if they met at least one of the following criteria: (1) they were regarded as landmark studies that made a substantial contribution to the development of knowledge regarding the relationship between screen time and childhood obesity; (2) they provided original evidence that has been consistently cited in subsequent literature; or (3) they offered important theoretical background necessary for understanding the evolution of research in this field.

The literature selection process involved a stepwise assessment of titles, abstracts, and full texts, guided by thematic relevance and contribution to the conceptual framework of the review. A total of 104 publications met the eligibility criteria and were included in the narrative synthesis.

Exclusion criteria comprised studies conducted exclusively in adults, publications unrelated to screen time, childhood obesity, or the mediating mechanisms linking these factors, non-peer-reviewed publications, conference abstracts without access to the fll text, and publications that did not meet the predefined eligibility criteria.

It should be emphasized that this narrative review focuses primarily on the potentially passive consumption of screen-based media for entertainment and educational purposes, and its indirect impact on the development of overweight and obesity among children and adolescents. It should be noted, however, that the latest screen media can increasingly support and promote physical activity, a point we have only touched upon; nevertheless, this constitutes a separate area of research that requires a separate study.

## 3. Screen Time as a Risk Factor for Overweight and Obesity

Today’s generation of children and young people is growing up in a highly digitalised environment. New media, such as social media, video games and apps, offer quick access to entertainment, knowledge and social interaction, and provide attractive, interactive ways to spend free time, clearly differing from passive traditional media such as television, radio, newspapers and films. Interactive media allow users to engage with them by creating and sharing personalized content, such as photos, videos or voice recordings, in line with their personal preferences [21]. The wide range of entertainment offered by new media means that most young people are constantly online, which manifests itself in so-called media multitasking, meaning that they use more than one form of screen-based media at the same time. This allows young people to watch television and be active on social media simultaneously. This behaviour is exhibited by 50% of the teenage population, whilst American statistics indicate that over half (60%) of young people aged 13–17 create a personal profile on more than one social networking site [22].

The evolution from traditional media to modern digital media has led to changes in screen usage patterns among children and young people [23]. In 2016, three-quarters of teenagers owned a smartphone, 24% reported being constantly online, and around 50% stated that they could not imagine life without a phone, which indicated media addiction [24]. Furthermore, there has been a significant decline in television viewing in favour of new, alternative devices and the possibilities they offer. Most of the children surveyed (85%) aged between 3 and 17 prefer to use a phone or tablet to watch films rather than a television [25]. Nevertheless, among children aged 8 and over, the average time spent watching television remains over 2 h a day, which is longer than recommended by the American Academy of Pediatrics (AAP) [26].

In 2020, the COVID-19 pandemic was one of the greatest disruptions to everyday life. In the spring of 2020, remote learning was introduced, shifting the education of 1.5 billion children online. Since then, access to digital devices and the internet, as well as the number of hours spent using media, have risen sharply among children and young people worldwide. During this time, the internet enabled many areas of life to function smoothly and, most importantly, ensured the continuity of education. The pandemic situation meant that interactive media became a conduit for interpersonal relationships, a way of spending free time with peers, and the only ‘window to the world’ [23].

As data from 2023 shows, the majority of households (97%) with children aged 0–18 had internet access, representing an increase of nearly 10% compared with data from the Central Statistical Office (GUS) for 2019 [27]. Data from the last quarter of 2024 indicate that nearly 2.7 million children (83%) aged 7 to 14 used the internet regularly. Over half, i.e., 58%, of this group use messaging apps permitted from the age of 13, such as TikTok or Facebook. In contrast, the group of older children (aged 15–18) using the internet at that time was smaller, numbering 1.49 million [28].

The most frequently used social media platform during lockdown was TikTok, followed by Facebook and Instagram, as well as video games, through which children maintained relationships and shared their free time, suggesting they had become accustomed to spending time in this way [8].

Modern technologies have permeated many areas of everyday life, including entertainment, communication and education, making it difficult to maintain a clear distinction between screen time allocated to different aspects of life [29,30]. The pervasive digitalization has a significant impact on the development and health of children and young people. Many recent studies have focused on screen time and its consequences for sleep, mental, emotional and physical health, including the development of overweight and obesity among children and adolescents [31].

In recent years, there has been an increase in overweight and obesity among children and adolescents. It is a fact that the primary determinants of overweight and obesity are an excess of calorie intake and insufficient physical activity [32]. Nevertheless, in recent years it has been shown that excessive exposure to screen-based media is a possible predictor of the development of obesity [33]. It is suggested that this association may have little clinical significance during childhood but significantly influences the development of obesity in later life [34]. It has been shown that obese children are predisposed to a higher incidence of cardiovascular disease, type 2 diabetes, and to remaining obese in adulthood, which in turn has significant health implications. It has been shown that adults with obesity have a higher risk of metabolic syndrome, stroke, and a higher risk of certain cancers of the breast or colon [32].

Numerous authors compare their research findings with the guidelines of the American Academy of Pediatrics (AAP), which has proposed a two-hour limit on screen time for children and adolescents aged 2–18 years as a preventive measure against the development of obesity [21]. However, a recent study by Wen et al. showed that every additional hour per week spent on screen time was associated with an increase in BMI in two-year-old children [35]. Nevertheless, Jong and colleagues demonstrated that as little as 1.5 h of screen time correlates with the development of obesity among children aged 4–9 years [36]. An international study by Braithwaite et al., involving 300,000 children, confirmed a 27% increase in the risk of obesity as a result of watching television for 1 to 3 h a day [37]. The results of these studies may form the basis for health organizations to establish new recommendations regarding media use, with the aim of reducing the risk of developing overweight and obesity.

It has been shown that excessive screen time during childhood and adolescence can influence metabolic risk factors associated with obesity, such as insulin resistance, plasma lipoprotein profile and liver enzymes. From the results of Sayin’s study, conducted among 108 obese children aged 10–15, we know that exposure to screen-based media for more than 5 h a day was associated with a higher HOMA-IR index, indicating impaired carbohydrate metabolism, as well as higher levels of aspartate aminotransferase (AST), alanine aminotransferase (ALT), and triglycerides (TG) [38].

The link between the rise in obesity and media exposure has been extensively studied for years. Hypotheses point to several mechanisms underlying the correlation between screen time and weight disorders in children, including sleep deprivation, an excess of calorie intake whilst using screens, and the simultaneous negative influence of unhealthy food advertising [33]. An additional factor contributing to the development of obesity driven by excessive digitalization is a decline in mood and depressive states, resulting in emotional, compulsive eating, as well as an increase in sedentary time at the expense of physical activity [39,40].

The physical effects of remote learning during the pandemic, when Polish children spent nearly 9 h a day sitting down, contributed to weight gain among pupils [41]. The World Health Organization reports that the lockdown period led to a weight gain of 2 kg in both children and adults [42]. At the same time as the lockdown, there was an increase in advertising for foods of low nutritional quality, characterized by high levels of salt, simple sugars and saturated fat, which have negative health effects and contribute to weight gain [43]. Furthermore, a 124% increase in food purchases was recorded, including highly processed takeaway food of low nutritional quality and high caloric density [44,45]. During the pandemic, the food industry quickly noticed the increased presence of children online and stepped up the development of online advertising and marketing of food products on the most widely used apps at the time, such as YouTube, Facebook and Twitter [46]. Research clearly shows that the products featured, even in a 30 s advert, significantly influence children’s dietary preferences and their choice of the featured brand [47,48]. Furthermore, new food marketing aimed at children often takes the form of entertainment or notifications from friends, which makes it even more difficult to distinguish it from the covert influence of advertising [49].

Research by Bród et al. indicates a significant link between excessive screen time among children and their dietary preferences. It was noted that among the children studied, there was an increase in the consumption of energy-dense snacks, drinks high in simple sugars and fast food, alongside a decrease in the consumption of fruit and vegetables [50]. In turn, Marazello and colleagues demonstrated that eating meals whilst watching television suppressed satiety signals, reduced conscious control over food intake and promoted an excess of consumed kilocalories [51], without any possible compensation in the form of reduced food intake for the remainder of the day [52]. An example of this can be found in the findings of Blass et al., who discovered that consuming a meal consisting of pasta and pizza whilst watching television for half an hour resulted in an additional 288 kilocalories, compared to eating these meals without watching TV. Similar findings regarding increased food intake were observed whilst playing video games (+80 kcal) or working remotely, involving writing and reading (+229 kcal) [53,54]. It has been shown that the feeling of satiety is indirectly linked to children’s BMI. This mechanism explains the fact that, as body fat increases excessively due to a surplus of kilocalories and unused energy, the concentration of leptin secreted by adipocytes rises. Furthermore, target tissues become resistant to the action of the hormone responsible for satiety, leading to the development of so-called leptin resistance. Consequently, children with an abnormal sense of satiety after a meal reach for more food, which inevitably leads to an increase in BMI and a vicious cycle of interrelated mechanisms [39].

## 4. Intermediary Mechanisms Linking Screen Time to Being Overweight and Obesity

### 4.1. Sleep Disorders

Sleep is essential for the normal growth and development of children and adolescents. It has been demonstrated that prolonged use of screen-based media has a negative impact on the quality and duration of children’s sleep [55]. This association is confirmed by a literature review by Hale et al., in which 90% of 67 studies showed a correlation between excessive screen time and a deterioration in sleep parameters, such as duration and quality [56].

The possible impact of the media on sleep may involve factors such as the emission of artificial light with a short wavelength (blue light) and the associated hormonal issues, as well as the direct delay in the time taken to fall asleep resulting from media use. Furthermore, exposure to stimulating content in the evening and the resulting mental, emotional or psychological stimulation, causing difficulties in falling asleep and maintaining sleep, may also explain the hypothetical mechanisms underlying the correlation between screen time and sleep [21,57]. Nevertheless, each of these possible relationships requires separate examinations to better understand the impact of screen time on sleep.

Recognizing the vital importance of sleep, members of the American Academy of Pediatrics (AAP) and experts from the National Sleep Foundation have drawn up recommendations on the appropriate amount of sleep for children and young people aged 0–18, with the aim of promoting children’s healthy development and well-being. According to the AAP, children aged 3 to 5 should sleep for 10–13 h, and those aged 6 to 12 should spend 9–12 h a day sleeping. Teenagers aged 13 to 18, on the other hand, should sleep regularly for 8–10 h a day [58,59]. Furthermore, the World Health Organization (WHO) has developed additional guidelines regarding the regulation of screen-based media use for children aged 0–5 years. Children under 1 year of age are not recommended to be exposed to screens at all. Children between 1 and 5 years of age should not be exposed to screens for more than one hour [12].

### 4.2. Blues Light Exposure and Sleep

The human biological clock is divided into two components: the central clock, located in the suprachiasmatic nucleus (SCN) situated in the anterior hypothalamus above the optic chiasm, and the peripheral clock, which is present in virtually every tissue and organ. Sunlight synchronizes the human biological clock, i.e., the circadian rhythm [60]. In turn, the synchronization of the peripheral clock arises from the integration of the central clock and external factors such as physical activity, sleep, and, above all, mealtimes [61]. The SCN, in response to light stimuli received by light (melanopsin) receptors located in the retina of the eye, synchronizes the biological clock, thereby regulating virtually every physiological process in the body. The circadian rhythm and hormones dependent on it play a key role in glucose and lipid metabolism, as well as in the endocrine, immune and reproductive systems, and in the pathophysiology of diabetes and obesity [62,63].

Melatonin is a hormone produced by the pineal gland, responsible for regulating the circadian rhythm and sleep; in particular, it influences the sensation of sleepiness and the maintenance of sleep at night [64]. The secretion of this hormone exhibits a strong circadian rhythm. The peak in hormone concentration is estimated to occur in the evening around 9 p.m. or approximately 2 h before the usual bedtime, with a sharp drop at 8 a.m. or before the usual wake-up time. Blue light, with a short wavelength, emitted from the screens of electronic devices, particularly smartphones and tablets held close to the face, is significantly more effective at inhibiting the secretion of endogenous melatonin than daylight (which has a long wavelength). This results, among other things, in a delay in the time of falling asleep and, consequently, a disruption of circadian rhythms [65,66].

Furthermore, it has been observed that children are more sensitive to the light emitted by electronic devices than adults, due to age-related changes in the eye and the larger size of the pupil. A larger pupil allows for faster transmission of blue light through the retinal ganglion cells, making it more susceptible to the suppression of melatonin secretion. Findings from recent studies indicate that in pre-pubertal children, compared to post-pubertal adolescents, melatonin secretion in response to blue light exposure is more significantly suppressed [67,68]. These findings may explain the current WHO recommendations regarding the complete avoidance of screens by infants and children.

Numerous studies indicate that exposure to blue light emitted by screens on devices such as televisions, computers and smartphones during the day and in the evening causes a delay in the time taken to fall asleep, reduces the duration of night-time sleep, and leads to poorer sleep quality and fatigue the following day [69,70]. From Vijakkhana’s research, we know that evening exposure to screen-based media, in 208 infants monitored at 6 and 12 months of age, resulted in a reduction in night-time sleep duration at 12 months, compared to those not exposed to screens at that time, in both age groups. Furthermore, a study of 12-month-old infants exposed to screen media after 7 p.m. showed a 28- or 46 min reduction in night-time sleep duration on weekdays [71]. Cheung and colleagues reached similar conclusions, confirming that infants and young children aged 0.5 to 3 years who had been exposed to a touchscreen during the day slept for shorter periods at night and showed a delayed bedtime, compared with children of the same age who had not been exposed to a screen [72].

### 4.3. Media Consumption at Any Time of Day and Sleep

Hysing and his team demonstrated a link between screen-based media consumption during the day and the duration of sleep at night. In a study of nearly 10,000 Norwegian teenagers aged between 16 and 19, it was noted that screen time among teenagers during the day exceeding 4 h was associated with an increased risk of reduced sleep duration at night (less than 5 h) and a lower likelihood of sleeping for 7 to 8 h, which would result in a daily sleep deficit of approximately 2 h [73]. Varghese linked difficulties falling asleep to exposure to screen-based devices and social media (Facebook, YouTube) in a group of 3172 Italian adolescents aged 11 to 15. Data from a meta-analysis of 20 cross-sectional studies indicate a 53% higher risk of poor sleep quality in the group of adolescents who were regularly exposed to electronic device screens immediately before bedtime [57].

Nevertheless, conclusions have been drawn regarding the independent association between screen time and sleep. Continex demonstrated that screen time exceeding 5 h per day was associated with a higher risk of sleep disturbances and difficulty falling asleep, compared with children who spent 1 h on screens during the same period [74]. Similarly, another study demonstrated a twofold higher risk of reduced sleep quality among more than 4191 Lithuanian children aged 14 to 16 who used media during the day [75].

It has been shown that endogenous melatonin levels are suppressed not only during exposure to blue light, but also when cortisol levels rise [76]. Cortisol, also known as the stress/action and dawn hormone, is produced by the adrenal cortex, which is first stimulated by adrenocorticotropic hormone (ACTH). Its highest concentration is observed in the morning, approximately half an hour after waking, whilst the lowest concentration is recorded in the evening and in the middle of the night, which demonstrates its opposite effect to that of melatonin, the ‘sleep hormone’ [65]. The use of screens, including emotionally engaging video games on a computer or touchscreen, as well as exposure to graphic scenes intended for adults whilst watching television, affects physiological and psychological arousal, including cortisol production. It has been shown that the unnatural level of sensory stimulation from the media results in reduced feelings of sleepiness in the evening, delayed sleep onset [65,77], anxiety about falling asleep on one’s own, and possible night-time awakenings [78]. It has been shown that cortisol levels are significantly higher in people with sleep disorders than in those who sleep for an adequate duration [65]. Considering this, the American Academy of Pediatrics recommended in 2016 that families ensure their children aged 2 to 5 watch only high-quality, age-appropriate programs for a maximum of one hour per day [79].

Furthermore, there is evidence to suggest that even attempting to fall asleep near a small screen or in a bedroom with a television lead to reduced sleep duration and poor-quality sleep. This conclusion was presented by Fable and his co-authors in a study conducted among 2048 pupils in Year 4 and Year 7, which found a reduction in total sleep time of over 20 min when sleeping near a smartphone screen, compared to children who slept without a phone, regardless of whether there was a television in the bedroom. Sleep duration was also reduced by 18 min for children who slept in a room with a television, compared to children who did not fall asleep in a bedroom with a TV [80]. It has been shown that sleeping near a small screen resulted in a perceived lack of rest the following day, as reported by respondents [81]. Among American children, it has also been shown that poorer sleep quality was associated with leaving electronic devices switched on in the bedroom whilst sleeping [82]. This association can be attributed to audible notifications received on smartphones, such as text or voice messages, and the associated light emitted from the LEDs and screens of electronic devices held close to the face, which, as explained earlier, inhibits the secretion of endogenous melatonin more effectively than television light, which diminishes with distance [80].

Sleep plays a key role in maintaining physical and mental health. It not only serves a restorative function but also plays an important role in regulating metabolism, hormonal balance and appetite control, and influences the body’s immune functions [83]. Numerous studies indicate a link between short sleep duration and an increased risk of metabolic disorders such as type 2 diabetes, hypertension, certain cancers and depressive disorders. Furthermore, a few recent studies point to sleep deprivation as a modifiable risk factor for the development of overweight and obesity in children and adolescents [84].

A clear causal link between sleep deprivation and the development of obesity in chil-dren remains difficult to establish, and the current literature suggests only possible mechanisms underlying this correlation. One of these is the nocturnal secretion of neuroendocrine hormones such as leptin, ghrelin, insulin, cortisol and melatonin, which are involved in the regulation of appetite, carbohydrate metabolism and sleep–wake patterns [85].

It has been shown that, in addition to regulating the circadian rhythm, melatonin has a preventive effect on the development of diabetes and obesity. Sleep deprivation can have a negative impact on glycemic control, as measured by glycated hemoglobin (HbA1c), and lead to increased fasting glucose and insulin levels. Excessively high levels of these blood parameters are associated with reduced tissue sensitivity to insulin and the development of insulin resistance, and in the long term, type 2 diabetes [85,86]. Although the mechanism of hormonal regulation during sleep is not fully understood, most scientists point to the beneficial effect of melatonin on hormonal balance, and furthermore, to its antagonistic effect on insulin. It has been shown that people with type 2 diabetes have lower serum melatonin concentrations. Furthermore, it is a fact that, in addition to its role in promoting and maintaining sleep, melatonin exhibits potent antioxidant activity. Its peak concentration, which occurs between 4 and 8 a.m., stimulates the activation of other endogenous antioxidants, such as glutathione peroxidase and catalase. This is significant for people with type 2 diabetes, in whom systemic oxidative stress, exacerbated by hyperglycemia, plays a significant role in the pathogenesis and progression of the disease. The multifaceted, beneficial effects of melatonin in the body extend beyond its role in promoting sleep, which may provide a rationale for interventions aimed at improving sleep [87].

**Figure 1:**
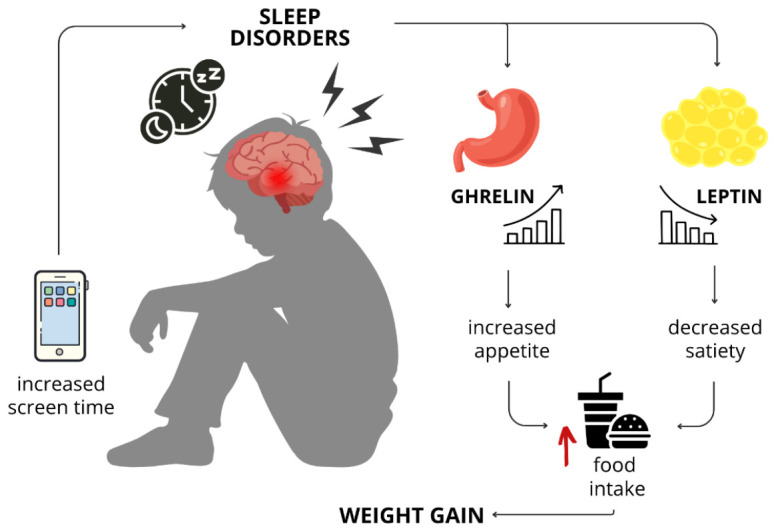
The impact of increased screen time on sleep disturbances and the hormonal regulatory mechanism responsible for controlling food intake.

As a result of reduced melatonin secretion following sleep disturbances, levels of cortisol—a hormone with an opposing effect—rise. Persistently high levels of this stress hormone in the blood have been linked to higher fasting blood glucose and insulin levels, reduced tissue sensitivity to insulin, obesity and type 2 diabetes. A study by Raikoonen, conducted among 282 children aged 8 who slept for less than the 7.7 h per day recommended by the AAP, compared with peers sleeping an average of 7.8–9.3 h per night, showed higher cortisol concentrations upon waking and throughout the day [88]. Research by Kim et al. has linked sleep deprivation to an increased HOMA-IR index, indicative of insulin resistance, metabolic problems, disturbances in the secretion of ghrelin and leptin, and reduced melatonin levels. Increased ghrelin secretion and lower leptin levels following sleep deprivation lead, respectively, to increased appetite and feelings of hunger, as well as reduced satiety after a meal, which results in overeating and weight gain (Figure 1) [89]. Another study, in which participants’ insulin levels were measured in the morning after 4 h of sleep compared to 8 h of sleep, showed a statistically significant increase in pancreatic hormone levels [90].

Recent epidemiological studies confirm a link between sleep deprivation in school-age children and low leptin levels [91]. A study conducted on 12 young men aged 22, whose sleep was restricted over two nights, resulted in an 18% reduction in plasma leptin secretion, a 28% increase in ghrelin, and a greater subjective sense of hunger and increased appetite, by 23% and 24%, respectively [92]. The results of Sayin’s study indicate that sleep deprivation influences metabolic factors associated with the development of obesity. Children aged 10–13 who slept for less than 9 h per night had significantly higher fasting insulin concentrations and HOMA-IR indices, as well as lower levels of HDL cholesterol, compared with children who slept for 9 to 10 h and >10 h per night—the amount recommended by the AAP [38].

Importantly, insufficient sleep relative to individual needs and age, and the associated reduction in melatonin secretion, were linked to increased feelings of tiredness and sleepiness during the day, as well as reduced motivation to engage in physical activity. Furthermore, low leptin levels following sleep deprivation and the resulting reduced feeling of satiety predispose children and adolescents to the development of overweight and obesity [93]. Similar findings are confirmed by a meta-analysis of 11 longitudinal studies involving 25,000 young people [94].

Martinez et al. demonstrated an association between getting sufficient sleep in line with WHO and AAP recommendations and lower weight gain among 229 children aged between 8 and 10 years over a two-year follow-up period [95].

The endocrine function of the lateral hypothalamus plays a key role in regulating the sleep–wake cycle, controlling hunger and satiety, and maintaining energy balance. This region contains glucose- and leptin-responsive neurons that produce hypocretin 1 (orexin A). Orexin A influences sleep, appetite and energy metabolism. When glucose levels fall, hypocretin neurons increase their activity, which leads to increased food intake. Conversely, when leptin levels rise after a night’s sleep, hypocretin neurons are inhibited, resulting in reduced food intake. Sleep deprivation and the associated lower leptin concentration significantly increase hypocretin 1 activity, leading to increased appetite and prolonged wakefulness. Furthermore, orexin A neurons increase alertness and physical activity; however, when sleep duration is reduced, the promotion of physical activity and energy expenditure is impaired. This neurological aspect may partly explain the link between sleep and the development of obesity [83].

Currently, the most commonly studied sleep parameter affecting increases in BMI in children is sleep duration. However, the time at which children fall asleep is also important [96]. Morrissey and colleagues report that later bedtimes on school days are strongly correlated with weight gain among children aged 7 to 10 years [97], whilst no significant correlation was found between wake-up time and the risk of obesity [98]. The results of studies conducted on children aged 8 to 17 confirm the link between late bedtimes and the development of overweight and obesity, regardless of sleep duration [99,100].

It has been observed that delaying bedtime at weekends also increases the likelihood of being overweight and obesity among children and adolescents [101]. In turn, Wing and colleagues demonstrated that sleep catch-up involving 5159 nine- and ten-year-old children at weekends and during school holidays may have a preventive effect on BMI increase. Nevertheless, the hypothesis regarding compensatory sleep at weekends in relation to inhibiting the development of obesity is inconsistent and requires further prospective and interventional studies [102].

### 4.4. Sleep Deprivation and Unhealthy Eating Habits

Another hypothetical mechanism underlying the development of overweight and obesity among children and adolescents, linked to sleep deprivation and changes in circadian rhythms, is the hormone-driven preference for unhealthy, high-calorie foods. A study by Chaput et al., involving 5777 children aged 9–11, reports that reduced sleep and later bedtimes encourage spending free time in a seated or lying position in front of a screen, and also translate into unhealthy eating patterns [103]. Sleep that is too short relative to requirements predisposes children to the consumption of high-energy fast-food snacks, rich in salt, sugar and fat, as well as sweet fizzy drinks, whilst concurrently reducing the consumption of fruit and vegetables among children [104]. Children’s and adolescents’ use of interactive media in the evening delays the time they fall asleep, which not only reduces the total amount of sleep due to school routines, but may also predispose them to behaviours associated with the development of obesity, such as spending longer periods eating or reduced physical activity [105]. Furthermore, it has been shown that eating sugar-rich foods late in the evening leads to a delay in bedtime and poor sleep quality. Pietrov and his team found that pre-school children aged 2 to 4 who slept for less than 11 h—which is shorter than the WHO’s recommendation for this age group—were more likely to reach for high-energy snacks rich in saturated fat and protein [106]. Wilson and Hermes, along with their teams, also report that insufficient sleep encourages the consumption of larger quantities of highly processed foods rich in saturated fats, a higher intake of simple rather than complex carbohydrates, and foods with a high glycemic index [107,108]. Regular consumption of foods with a high glycemic index and glycemic load is associated with an increased risk of developing type 2 diabetes, heart disease, overweight or obesity. The results of a meta-analysis of 9 randomized controlled trials, involving 1065 children and adolescents, showed that excessive consumption of foods with a high glycemic index (GI) and glycemic load (GL) was associated with higher levels of insulin resistance (HOMA-IR) and triglycerides (TG), compared to a dietary strategy based mainly on foods with a low GI and GL [109].

An experiment by Beebe and colleagues also confirmed that dietary preferences conducive to the development of obesity were evident following insufficient night-time sleep among healthy teenagers aged 14–16. Among the participants, after five days of 6.5-h night-time sleep compared to 10-h sleep, a greater tendency to reach for foods with a higher glycemic index and load was observed, particularly desserts and sweets, along with a tendency towards higher intake of kilocalories and carbohydrates, without significant differences in protein and fat intake [110]. Among 441 children whose sleep was restricted by one hour, compared to a control group, a higher intake of foods rich in simple carbohydrates and sweetened drinks was observed [111]. Similar conclusions were drawn by Spiegel et al. in a study conducted on 22-year-old men whose sleep was restricted to 6.5 h per night for two nights. Following this experiment, the preferred foods were characterized by high calorie density and a high content of simple carbohydrates, compared to the control group [92]. The link between short sleep duration and cravings for specific foods may be explained by the cholinergic–dopaminergic reward pathway, which is activated by ghrelin—a hormone whose levels rise abnormally following insufficient sleep. Night-time sleep deprivation in adolescents is associated with reduced sensitivity of the reward center, which in turn may influence the seeking of rewards during the day and night in the form of sweet foods and drinks, which cause the release of dopamine in the reward system, triggering the desired feeling of satisfaction and happiness [112].

The study by Ramirez-Contrenas notes a correlation between insufficient sleep and going to bed later, and a preference for high-carbohydrate foods and a higher BMI among children, which, as we know from the same study, is linked to a deterioration in sleep quality and a reduction in total sleep time. Furthermore, among children who delay their bedtime, a routine skipping breakfast was observed, in order to gain some extra screen time [113]. On the other hand, skipping the first meal, as confirmed by several studies, is associated with a higher risk of developing overweight and obesity in children and adolescents [114].

Although many studies have noted a correlation between bedtime, sleep duration and dietary preferences—which indirectly increase the risk of developing overweight and obesity among children and adolescents—there is a need for further cohort studies and randomized intervention trials using objective methods of sleep measurement and validated food frequency questionnaires and well-being following insufficient and adequate sleep. This would enable a more reliable determination of the causal relationship between sleep and a preference for highly processed foods as well as the identification of the biological mechanisms playing a significant role in this relationship.

The most important studies examining the relationships between screen exposure, sleep disturbances, and overweight/obesity risk in children and adolescents are summarized in Table 1.

### 4.5. Mental Disorders: Increased Levels of Stress, Anxiety, Restlessness, and Emotional Eating

Excessive stress and emotional disorders in children, particularly in adolescents, have a significant impact on their overall development [115]. Data from the Health Behaviour in School-Aged Children (HBSC) study conducted across Europe, Central Asia, and Canada among children aged 11, 13, and 15 indicate an increase in mental health problems, such as stress, anxiety, depression, and issues related to self-esteem among young people [116]. A growing body of research indicates that one of the key contributing factors to this phenomenon is excessive screen time, particularly in the context of social media use, computer gaming, and the consumption of television and video content [19,117,118]. Many researchers suggest that continuous exposure to digital stimuli, combined with insufficient sleep and reduced physical activity, manifests as disturbances in emotional functioning among children and adolescents, and consequently contributes to weight-related disorders [18,20].

It is widely proposed that three principal pathways explain the association between screen time and impaired mental well-being among young people. Firstly, time spent using digital technologies may limit engagement in alternative social and cognitively enriching activities (displacement hypothesis) [119]. Furthermore, exposure to diverse and idealized content, together with children’s engagement with the digital environment—particularly social media—may increase dopamine release in the brain, activating the reward system, which in the long term may predispose individuals to addictive behaviours and a consequent reduction in direct interactions with peers and family (social isolation) [120,121]. Additionally, excessive use of electronic devices, especially before bedtime, may disrupt circadian rhythms, and insufficient sleep as well as poor sleep quality are strongly associated with mental health problems, including anxiety and depression, in the pediatric population [122,123]. However, these underlying mechanisms remain insufficiently elucidated, and findings across studies are inconsistent [124]. According to Jensen et al., the use of digital technologies by young adolescents is associated with little or no increase in the severity of mental health symptoms [125]. In contrast, Hampton and colleagues emphasize that it is the lack of access to digital media, rather than its use per se, that may contribute to social isolation and negatively affect young people’s mental health [126]. Similarly, a study conducted by Orben et al. challenges prevailing assumptions regarding the detrimental effects of digital technologies on mental well-being in young populations, suggesting that this relationship is considerably more complex and multifactorial [77].

Nevertheless, a substantial body of evidence demonstrates a positive association between these phenomena. Findings from a large population-based study conducted by Twenge and Campbell indicate that increased screen time (>1 h per day) is associated with lower levels of psychological well-being, including higher levels of stress, reduced life satisfaction, and a greater prevalence of symptoms of depression and anxiety among children and adolescents aged 2 to 17 years [127]. Babic et al. similarly reported that recreational screen time is negatively associated with changes in physical self-perception and mental well-being in adolescents, whereas screen time related to non-recreational purposes (e.g., educational use) was not significantly associated with the occurrence of mental health symptoms [128]. Comparable findings have been reported in several systematic reviews [118,129,130]. In contrast, more consistent evidence has been documented regarding the association between screen time and depressive symptoms, particularly among adolescents [131,132,133]. However, it should be noted that much of the available evidence is subject to certain limitations. To date, the majority of studies have been cross-sectional in design and therefore do not allow for the assessment of causality or directionality in the observed relationships [129,134]. Consequently, there is a clear need for further well-designed cohort and experimental studies in this area.

When discussing the emotional well-being of children and adolescents in the context of screen time and digital media, it is worth referring to the psychological phenomenon known as FOMO (Fear of Missing Out), which has been extensively studied in recent years. It refers to a persistent need among children and adolescents to remain constantly engaged on social media, driven by concerns about missing important events or interactions with peers when they are not active online [135]. Fear of Missing Out (FOMO) is characterized by the experience whereby a lack of online activity may trigger anxiety, unease, a sense of isolation, and fear of social exclusion. Many young people, observing peers who share significant moments or notable achievements on social media, may experience feelings of inadequacy [136]. Although research on FOMO remains relatively recent, particularly in the context of children and adolescents, it already represents an important area of psychological research, emphasizing the impact of social media on mental health and social functioning in young populations [137].

There is a limited number of studies that clearly elucidate the mechanisms through which screen time, associated with stress and mental health disorders in early childhood and adolescence, correlates with the development of overweight and obesity. The lack of such data limits a comprehensive understanding of the underlying biological and behavioural pathways.

**Figure 2:**
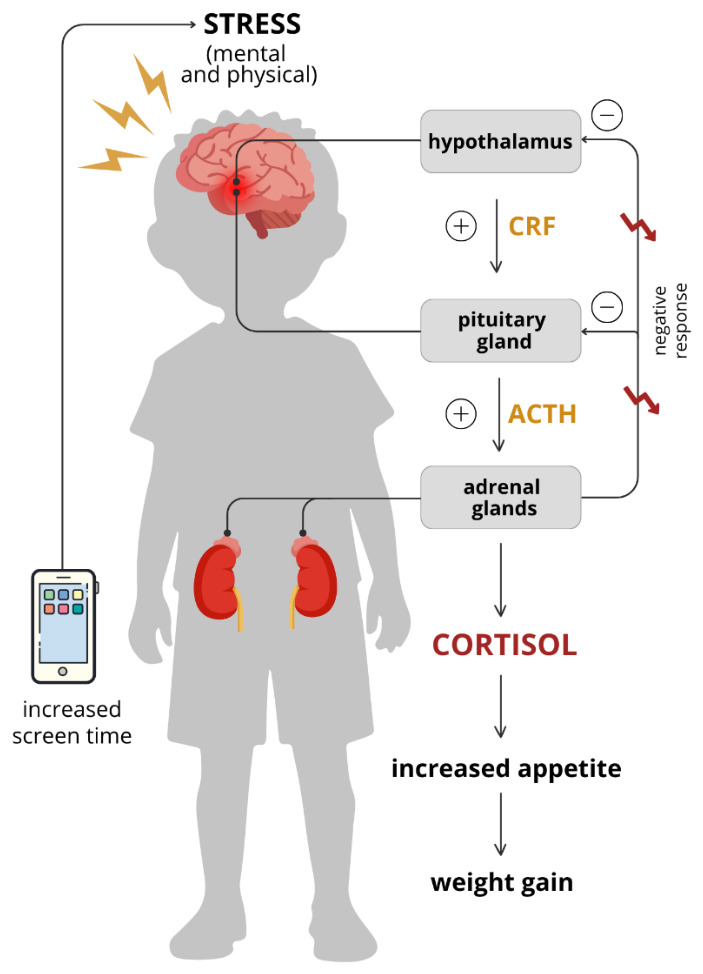
Dysfunction of HPA axis regulation caused by increased screen time and stress. Red downward arrows indicate disruption (dysregulation) of HPA axis function associated with increased screen time. CRH—corticotropin-releasing hormone, ACTH—adrenocorticotropic hormone.

However, according to current knowledge, stress-whether acute or chronic-activates the hypothalamic-pituitary-adrenal (HPA) axis, which is involved in the regulation of body weight [138]. During continuous exposure to stressors, often triggered by increased screen time (e.g., anxiety related to social media use, exposure to negative content, or stress associated with social isolation), the hypothalamus continuously releases corticotropin-releasing hormone (CRH), which stimulates the secretion of corticotropin (adrenocorticotropic hormone, ACTH) from the anterior pituitary gland, ultimately leading to increased cortisol production by the adrenal glands [139]. Under physiological conditions, elevated cortisol levels are regulated via a negative feedback mechanism (involving the hypothalamus and pituitary gland), allowing the body to return to homeostasis once the stressor has subsided. Excessive activation of the HPA axis may lead to dysregulation of this feedback system, resulting in sustained overproduction of cortisol (Figure 2) [140].

It is suggested that dysfunction in the regulation of the HPA axis may contribute to the development of overweight and obesity; however, the causal relationship between these factors remains unclear. According to scientific reports, chronically elevated cortisol levels are associated with increased appetite and a greater tendency toward overeating [141]. Furthermore, they may promote the long-term accumulation of visceral fat by facilitating the differentiation of preadipocytes into mature adipocytes (fat cells) [142]. It also appears that glucocorticoids impair insulin signaling at both peripheral and central levels, while elevated cortisol levels enhance glucose production in tissues (gluconeogenesis). This may predispose individuals to the development of insulin resistance and, consequently, to increased energy intake and the development of overweight or obesity [142,143].

The pediatric literature does not provide a substantial body of evidence that unequivocally confirms the mechanisms described above. Most studies are cross-sectional in design and focus primarily on adults and animal models [139]. However, Hillman et al. reported that obesity is correlated with increased activity and reactivity of the HPA axis in adolescent girls. Furthermore, these findings suggest that as the degree of obesity increases, there is a reduction in daytime cortisol levels and an increase during the night [144]. These data are consistent with the findings of Ruttle et al. [145]. Under physiological conditions, cortisol levels are lowest at night, increase prior to waking, and rise sharply upon awakening [146]. An observational study conducted by Wallenius et al. also demonstrated that time spent using digital media may influence cortisol regulation in school-aged children. Children who spent at least three hours per day using screens exhibited a reduced cortisol awakening response, reflected by a diminished increase in cortisol levels one hour after waking. In contrast, children who spent less than three hours per day using media, or did not use them at all, exhibited a normal morning rise in cortisol [147]. Disruptions in cortisol regulation have also been associated with social media activity. A study conducted among adolescents aged 12–17 years found that having a greater number of online friends was correlated with elevated daily cortisol concentrations [148].

It is proposed that stressful experiences in early childhood may influence the risk of subsequent obesity through multiple biological pathways [149]. Therefore, future experimental studies are needed to further elucidate the relationship between increased screen time, stress, cortisol levels, and eating behaviours, to fully understand their role in the development of weight gain in children and adolescents.

As mentioned earlier, screen time is closely linked to social media use. Children and adolescents, using various types of electronic devices, are increasingly spending time on social media (SM) platforms, which have become a primary source of entertainment and information, as well as a means of peer interaction. The question of the direct impact of social media on the mental well-being of young people remains a topic of ongoing scientific debate. Some studies indicate a lack of significant evidence confirming an association between the use of social media platforms and mental health symptoms in children and adolescents [21,150]. Further analyses describe the potential benefits of moderate social media use [21], whereas other data suggest contrasting findings [151,152]. A longitudinal study analyzing developmental windows of susceptibility to social media use during adolescence identified an association between more frequent social media use and reduced life satisfaction [153]. Furthermore, numerous reviews have shown that young people who spend more time on social media platforms such as Facebook or Instagram may experience depressive symptoms, including higher levels of anxiety and stress [154,155]. This phenomenon is often attributed to social comparison, whereby young people begin to compare their lives with the idealized representations presented by other users, resulting in feelings of inadequacy, envy, or reduced self-esteem [156]. Consequently, children and adolescents may exhibit signs of social isolation and related emotional disturbances [157]. The discrepancies observed across studies may be attributed to methodological differences, including variations in the definition and assessment of social media use, as well as differences in the characteristics of the study populations. Furthermore, growing evidence suggests that the impact of social media on mental health is influenced not only by the duration of use but also by the nature of online engagement, the purpose of use, and individual user characteristics. In addition, the predominance of observational study designs limits the ability to establish causal relationships and raises the possibility of reverse causality.

Difficulties in emotional regulation may frequently lead to maladaptive eating behaviours, particularly emotional eating (EE) [158]. This is defined as the consumption of food in response to intense emotional experiences, regardless of physiological hunger [159]. Eating in response to negative emotions is often associated with the selection of energy-dense, ultra-processed foods, including products containing added fat and/or refined carbohydrates, which provide immediate pleasure and satisfaction, effectively diverting attention from negative emotional states [160]. This is because the consumption of so-called “comfort food” activates brain regions (primarily the reward system) that are central to the experience of pleasure [161]. A cross-sectional study conducted in India on a sample of 100 children aged 8–10 years revealed a positive association between increased screen time and emotional overeating [39].

A significant body of research supports the hypothesis that emotional eating may contribute to weight gain and the development of childhood overweight and obesity [160]. A study by Sanchez et al., conducted as part of the Chilean GOCS (Growth and Obesity Chilean Cohort Study), analyzed the relationship between eating behaviours in children aged 7–10 years and their body mass index (BMI). It was observed that a tendency to eat in emotional situations and in response to environmental cues was associated with higher BMI values in children [162]. Furthermore, the findings of a large observational study suggest that emotional impulsivity—that is, a tendency to act under the influence of strong emotions—is associated with higher BMI in European adolescents. This may indicate an indirect association between emotional eating and obesity [163]. Nevertheless, there is also evidence that does not support a direct association between EE and weight-related disorders in young people [164]. This may be attributed to methodological heterogeneity, differences in study population characteristics, or individual factors influencing body weight. Therefore, further research in this area is warranted.

A summary of the principal studies discussed in this section is presented in Table 2.

### 4.6. Reduced Physical Activity

Physical activity is recognized as a non-pharmacological preventive factor for many diseases, such as high blood pressure, diabetes and cancer. At the same time, physical activity is essential for maintaining mental health, supports cognitive function, helps maintain a sense of well-being, and it can have a preventive effect and alleviate the symptoms of depression and anxiety. Furthermore, regular exercise helps maintain normal bone density and body weight [165,166].

As data from the UNICEF report indicate, physical activity levels among children and young people remain consistently low, whilst the amount of time spent sitting—often in front of screens—is on the rise [167]. This is also confirmed by the study conducted by Zawadzka et al. in all voivodeships in Poland on behalf of the Minister of Sport and Tourism [168]. Since the start of the coronavirus pandemic lockdown, the results of numerous observational studies have indicated longer screen time among children, significantly exceeding the recommendations of the American and Canadian Pediatric Societies, which call for a maximum of two hours of screen time [167,169,170].

Considering the multidimensional, beneficial effects of physical activity on health, the World Health Organization (WHO) recommends that young people aged 5 to 17 should spend an average of 60 min each day on moderate- or vigorous-intensity physical activity. In addition, at least 3 days a week should be devoted to high-intensity aerobic exercise and activities that strengthen muscles and bones. At the same time, the WHO recommends that children in this age group minimize the time spent sitting whilst using screen-based media for entertainment [165].

There is a possible link between device consumption and reduced activity, which is associated with the displacement of other activities, and the more interesting ways for children to spend their free time, which are screen media, without the consequences of stimulation. Furthermore, after school and extracurricular activities, such as tutoring, often lasting until late in the afternoon, children lack the energy for active play or the opportunity to go outside freely without supervision. Evenings spent using screens are often used as a remedy for boredom at home and engaging with peers. There has been a recent increase in the use of an additional electronic device as part of a routine before bed or as a false aid for insomnia. A large observational cohort study conducted as part of the Adolescent Brain Cognitive Development Study (ABCD) found that both excessive screen time (>4 h per day) and low physical activity (<12,000 steps/day) are independently associated with a higher BMI and an increased risk of being overweight or obese among American adolescents aged 10 to 14. More importantly, high levels of physical activity did not offset the negative impact of prolonged screen time on body mass index, just as low screen time did not compensate for a lack of physical activity [171]. Conversely, the results of a 2-week randomized clinical trial by Pedersen and colleagues demonstrated that limiting recreational screen time to ≤3 h per week resulted in an increase in time spent on physical activity by an average of 44.8 min per day among 86 children aged 8–9 years, compared with a control group of 95 children [172]. However, the intervention study cited involved a relatively small group of children and a short follow-up period, which limits the ability to assess long-term effects.

Although available scientific evidence suggests a potential correlation between screen media use and lower physical activity, as well as their association with the development of overweight and obesity, it does not allow for a clear determination of cause-and-effect relationships. The results suggest that both limiting screen time and engaging in regular activity are crucial for health, which could be a direction for future intervention studies.

### 4.7. Active Screen Time

It is widely recognized that interactive media have become a major influence, which has a real impact on the preferences and choices of children and young people, including their involvement in physical activity. Recently, the potential of new media has been recognized in promoting healthy behaviours, such as spending free time actively, accessing free health-related information provided by medical experts, and supporting large population groups in changing their health habits [173]. An additional benefit of using social media to encourage improvements in health and physical fitness includes, amongst other things, the opportunity to interact with peers during training sessions, emotional support, and supervision of the recovery process [174]. New technologies can promote an active lifestyle through animated games that require specific body movements to progress through levels, popular dance routines in the form of short social media videos that involve choreography and exercise, group fitness challenges, one-to-one sessions with personal trainers, or participation in e-sports [77,175,176]. Moreover, thanks to new smartphone apps developed in response to the reorganization of healthcare during the pandemic, parents of obese children can learn about the causes, symptoms and prevention plans aimed at reducing their children’s weight [177].

New technologies have the potential to promote physical activity; however, it is crucial that they serve merely as a complement to, rather than a substitute for, traditional forms of active outdoor recreation and interaction with peers or parents. There is a need for more randomized controlled trials in this area in the future to determine whether screen-based media can effectively help curb the rising tide of childhood and adolescent obesity [178]. Furthermore, the post-COVID reality also requires a focus on strategies to address excessive media consumption and the associated sedentary lifestyle [179].

## 5. Prevention and Intervention Strategies

The current generations of children, namely Generation Alpha and Generation Beta, born between 2010 and 2024 and between 2025 and 2039, respectively, are growing up in a rapidly changing environment saturated with interactive media, artificial intelligence and marketing of low-quality food, which may have a real impact on the health and food choices of the younger generation, and consequently on their overall health. In this context, it is essential to raise awareness among the younger generation and for parents to implement health-promoting behaviours at home, such as shopping together, physical activity or eating meals, as well as limiting screen time, and fostering healthy eating habits and health-promoting behaviours that will have a tangible impact on their current and future lives [179].

There is evidence to support the benefits of involving the whole family, as opposed to children alone, in potentially preventing the development of obesity; one reason for this may be the ability to provide support in overcoming the barriers that hinder the maintenance of healthy behaviours. In a study by Robson et al., participants took part in a 10-week cooking programme designed to reduce the consumption of meals eaten outside the home. The study showed that the proportion of family lunches eaten outside the home fell from 56% to 25% [180]. Following this line of thinking, future research could explore how the involvement of the whole family in intervention activities might contribute to more frequent physical activity at the expense of screen time.

The ‘Let’s Go! 5-2-1-0’ program can be helpful in tackling the growing crisis of childhood overweight and obesity. This tool, recommended for children and developed by the Barbara Bush Children’s Hospital at Maine Medical Center, recommends four healthy daily habits, such as eating five or more portions of fruit and vegetables, limiting screen time to ≤2 h, spending at least one hour on physical activity, and avoiding sugary drinks [181].

An attempt was made to demonstrate the effectiveness of the Let’s Go! 5-2-1-0 programme in a controlled pilot study conducted over a six-month period among 15 families with children aged 5–13. In addition to the intervention programme’s objectives, monthly joint 1–2 h cooking sessions, 1 h of joint physical activity, and health education sessions incorporating the 5-2-1-0 programme’s principles were implemented. The results of the study showed that the pilot intervention only affected knowledge of healthy eating habits; however, it had no significant impact on weight reduction (BMI), waist circumference, or behavioural improvement [182]. One of the most significant studies documenting the impact of Let’s Go! involved 12 communities in the state of Maine, including 800 families with children. After 4 years of observation, an increase in fruit and vegetable consumption among children was demonstrated (from 18% to 26%), an increase in the reduction in sugary drinks (from 63% to 69%), and an increase in health awareness from 10% to 47% [183].

Other tools for managing the weight of obese children include Bright Bodies and MEND (Mind, Exercise, Nutrition… Do It!). These are programmes based on a family-centred lifestyle intervention that encompasses education, physical activity and behavioural modification. Bright Bodies is designed for children aged 8 to 16 struggling with obesity (BMI above the 95th percentile for their age and gender), whilst MEND is for children aged 7 to 13. The therapeutic efficacy of Bright Bodies was confirmed by a one-year randomized clinical trial involving 170 overweight children and adolescents without diabetes. The programme significantly improved metabolic parameters, including increased tissue sensitivity to insulin and reduced blood insulin levels; a significant improvement in BMI and body composition was observed, including a reduction of 9.2 kg in total body fat compared with the control group receiving standard care [184]. A similar beneficial effect of the large-scale MEND environmental intervention was associated with an improvement in BMI in obese children and improved psychosocial outcomes [185]. One way of assessing the risk of developing obesity in children could be a pediatric version of the tool for assessing obesity in adults, developed by the Unraveling Obesity team. The tool was introduced to the market in 2022 and is available online, designed for children aged 5 to 12 and adolescents aged 13 to 19. It is based on the four pillars of clinical treatment for obesity, developed by the Obesity Medicine Association (OMA), as well as the OMA Pediatric Obesity Algorithm. The OMA Pediatric Obesity Algorithm is a clinical tool for the treatment of obesity, developed based on scientific evidence, supported by the literature and the clinical experience of practicing pediatricians treating obesity in infants, children and adolescents. Using Unraveling Obesity’s tools to conduct an assessment—at home under parental supervision or in a waiting room before a doctor’s appointment, for example—allows parents and healthcare professionals to access the results immediately, which can then be used to formulate treatment recommendations or refer patients to specialists who work with childhood obesity on a daily basis [179]. Although the tool is evidence-based, there is a lack of intervention studies to confirm its effectiveness in clinical trials.

Similarly, the tools, such as Let’s Go! 5-2-1-0 programme and Bright Bodies, are consistent with current recommendations for a healthy lifestyle and appear to have potential in the context of childhood obesity; however, they should not be regarded as standalone tools for the prevention and treatment of obesity. There is still a need for studies evaluating the effectiveness of these tools on a larger scale, over a longer duration and under comparative conditions.

It is imperative to prevent excessive media consumption and its indirect impact on the development of overweight and obesity among children and young people by adhering to the screen time guidelines developed by the American Academy of Pediatrics (AAP), which recommend a maximum of two hours of screen time per day. Numerous studies clearly indicate that regulating screen time promotes more effective time management, stabilizes daily routines and increases the likelihood of engaging in physical activity, which is crucial for the proper development and maintenance of healthy body weight in children [186] Padersen and colleagues presented significant findings in their study, indicating that limiting recreational screen time resulted in an increase of approximately 45 min per day in physical activity in the intervention group [172].

Certainly, it will be important to recognize and appreciate the vital role parents play in shaping the appropriate use of digital media and in striking a balance between screen time and other activities in children’s daily lives. In the post-COVID era, saturated with engaging interactive media (e.g., YouTube, TikTok), which, through their algorithms, effectively capture the attention of children and young people, it will be essential for parents to offer sufficiently interesting and engaging forms of physical activity that will effectively encourage children to be active at the expense of screen time. For example, these might include activities organized by local communities, such as inter-school competitions; access to playgrounds and sports pitches; training sessions with coaches at sports clubs, for example after school; or active travel to and from school, for example by bike, on foot, on roller skates or on a non-motorized scooter. This requires suitably safe and well-maintained cycle paths, which depend on parental involvement and the actions of the local community, as well as government initiatives and the development of spaces conducive to physical activity. Reducing screen time may be linked to mental well-being and a lower risk of anxiety, depression and sleep disorders, which are often associated with excessive use of digital media. Parental control over children’s screen time helps to increase the number of face-to-face family interactions and meaningful conversations. Such forms of contact play a key role in strengthening emotional bonds, developing communication skills and reinforcing a sense of closeness and belonging within the family unit, thereby reducing stress and anxiety in children.

Parental education and awareness, particularly among those with a higher socio- economic status and a level of education typical of European countries, are crucial for the early development of healthy physical activity habits in children. Introducing children to regular physical activity at an early age can help to establish healthy behavioural patterns later in life. It is important that, as they grow, children maintain a low level of sedentary behaviour, as encouraged by, among others, the ‘Let’s Go 5-2-1-0’ programme and the World Health Organization’s guidelines [12].

Furthermore, the medical community, and pediatricians in particular, can help to identify issues relating to excessive screen time during routine check-ups with their patients. Thanks to regular contact with children and their carers, they can observe worrying signs, such as sleep problems, difficulty concentrating, low mood, reduced physical activity or increased calorie intake which contribute to development overweight and obesity. Early recognition of such symptoms allows for preventive measures to be taken as quickly as possible, as well as for the family to be referred to appropriate specialists or for specific strategies to be proposed to limit screen time.

## 6. Limitations and Future Research

Despite the multifaceted discussion of the topic and the consideration of numerous mechanisms—behavioural, biological and psychological—this narrative review is subject to certain limitations that prevent the establishment of cause-and-effect relationships. Most of the studies we analyzed are observational in nature, which limits the ability to draw definitive conclusions.

A strength of our work is the comprehensive analysis of potential mechanisms linking excessive screen use with the development of overweight and obesity in the pediatric population, taking into account possible hormonal, metabolic and psychological aspects that may influence the pathogenesis.

About the development of overweight and obesity in the pediatric population, stronger scientific evidence supports the role of hormonal and metabolic mechanisms rather than reduced physical activity displaced by screen-based media. Although some studies suggest that screen time may replace active forms of leisure activity, this association is generally weak or moderate in our studies and requires further investigation in larger population groups. Furthermore, a growing body of evidence suggests that excessive use of screen-based media contributes to sleep disturbances and the disruption of the circadian rhythm, including hormonal secretion (e.g., melatonin, ghrelin, leptin, cortisol) [38,88,89,90,92], which indirectly contributes to excessive consumption of highly processed foods and weight gain. The current state of knowledge therefore indicates that the relationship between screen-based media and obesity is multifactorial, and that metabolic and hormonal aspects appear to play a more significant role in the development of overweight and obesity than the replacement of physical activity with screen time.

Future studies should be conducted on larger cohorts and focus on the use of appropriately validated methods for measuring physical activity, such as accelerometers. A significant proportion of existing research relies on self-reported physical activity measures provided by parents, which may be subject to memory errors, subjective assessment, and a tendency to provide answers that align with societal expectations.

Furthermore, it would be important in future to conduct research into the impact of screen time on overweight and obesity, broken down by gender, age group and the type of screen time, as these factors may have different effects. A few studies suggest that girls use social media and online interactions more frequently than boys, which, as mentioned in the review, may contribute to the development of depressive states [127], a decline in self-esteem, and dissatisfaction with appearance more so than the use of other media such as television. Boys, on the other hand, are more likely to engage with video content and play computer games, which may potentially affect levels of physical activity, sleep patterns and social behaviour [187,188]. Although the potentially different ways in which girls and boys use screens have not been described in detail—as this was not the focus of our discussion—we view this as a research gap in the present review and a topic that could be addressed in a new review. Focusing on the effects of different types of screen time consumption between genders and duration would enable the design of effective preventive and educational measures tailored to the needs of a given group.

Another promising area of research would be to evaluate the effectiveness of personalized preventive interventions such as the ‘Let’s Go! 5-2-1-0’ programme, Bright Bodies or MEND (Mind, Exercise, Nutrition… Do It!) Prospective and long-term studies would be particularly important, taking into account different age groups and programme implementation settings, as well as comparing the effectiveness of these models with other preventive interventions. These results could be used to develop effective, targeted strategies for the prevention of overweight and obesity, with implications for adult life.

## 7. Conclusions

**Figure 3:**
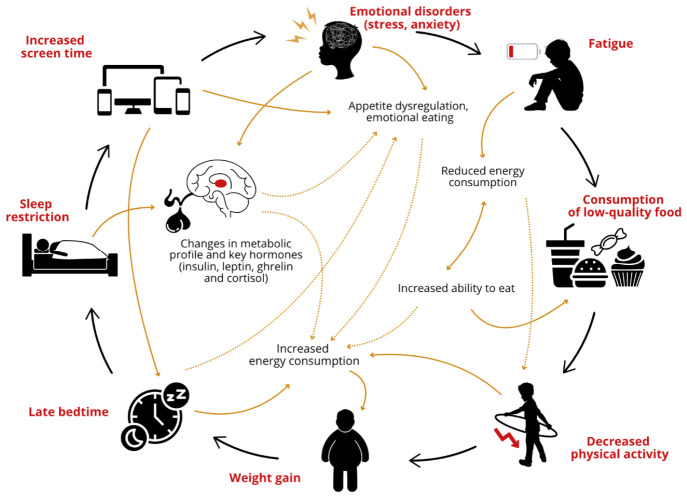
Interactions between increased screen time, sleep deprivation, mental health disorders, reduced physical activity and their impact on food intake and weight gain. Adapted with permission from Calcaterra et al., Nutrients 2023, doi: 10.3390/nu15224736 [84]. Created using Canva (https://www.canva.com, accessed on 26 March 2026).

The evidence reviewed highlights the ongoing digitalisation of younger generations, while also pointing to the potential consequences of excessive electronic device use, including sleep disturbances, mental health problems, and reduced physical activity. The mechanisms underlying these associations are complex, multidirectional, and likely interact with and reinforce one another. Consequently, they may indirectly contribute to a positive energy balance and an increased risk of overweight and obesity in children and adolescents (Figure 3).

Effective preventive strategies will require a comprehensive approach involving families, educational institutions, and healthcare systems, integrating medical, psychological, and educational expertise. At the same time, there is a need to develop, implement, and evaluate evidence-based diagnostic tools, as well as educational and intervention programmes aimed at promoting healthy sleep habits, regular physical activity, healthy dietary behaviours, and psychological well-being among children and adolescents. A holistic approach that addresses all of these factors is likely to be more effective in preventing overweight and obesity than strategies that focus solely on reducing screen time.

## Figures and Tables

**Table 1:** Summary of key studies examining the associations between screen time, sleep disturbances, and overweight/obesity risk in children and adolescents.

Authors (Year)	Study Design	Sample/Age Group	Screen Type	Main Outcome
Hale & Guan (2015) [56]	Systematic review	67 studies involving school-aged children and adolescents	Television, computers, video games, and mobile devices	Screen time was adversely associated with sleep outcomes in 90% of studies, particularly with shorter sleep duration and delayed sleep onset.
Vijakkhana et al. (2015) [71]	Prospective cohort study	*n* = 208 infants followed from 6 to 12 months of age	Screen media exposure (TV, DVDs/videos, computers, tablets, and mobile phones)	Evening screen media exposure was associated with shorter night-time sleep duration. Infants exposed to screen media after 7 p.m. had approximately 28 min less sleep at 12 months of age.
Hysing et al. (2015) [73]	Cross-sectional population-based study	*n* = 9846 adolescents aged 16–19 years	Computers, mobile phones, tablets, TV, game consoles, and Internet-based activities	Daytime and bedtime screen use were associated with an increased risk of short sleep duration, prolonged sleep onset latency, and sleep deficiency, with a clear dose–response relationship.
Falbe et al. (2015) [80]	Cross-sectional study	*n* = 2048 children (4th- and 7th-grade students)	Small screens (e.g., smartphones), TV, video/computer games	Sleeping near a small screen was associated with 20.6 fewer minutes of sleep per night, whereas the presence of a TV in the bedroom was associated with 18 fewer minutes of sleep.
Varghese et al. (2021) [57]	Cross-sectional study	*n* = 3172 adolescents aged 11–15 years	Social media (Facebook, YouTube) and electronic device use	Frequent use of social media and electronic devices was associated with increased sleep-onset difficulties. The strongest association was observed for YouTube use (OR = 2.00).
Fatima et al. (2015) [94]	Systematic review and bias-adjusted meta-analysis	22 longitudinal studies; meta-analysis of 11 studies (*n* = 24,821 children and adolescents)	Indirect (sleep duration)	Short sleep duration was associated with a more than twofold increased risk of future overweight and obesity.

**Table 2:** Summary of selected studies investigating the associations between screen time and potential mechanisms contributing to childhood obesity.

Authors (Year)	Study Design	Sample/Age Group	Screen Type	Main Outcome
Twenge & Campbell (2018) [127]	Cross-sectional population-based study	*n* = 40,337 children and adolescents aged 2–17 years	Overall screen time (cell phones, computers, electronic devices, electronic games, and TV)	Screen time > 1 h/day was associated with lower psychological well-being. Adolescents using screens ≥ 7 h/day had more than twice the risk of depression and anxiety compared with low users (1 h/day).
Babic et al. (2017) [128]	Longitudinal study	*n* = 322 adolescents (211 girls, 111 boys); mean age 14.4 ± 0.6 years	Recreational screen time (TV/DVD, computer, tablet/mobile phone) and non-recreational screen time	Increases in recreational screen time were associated with poorer psychological well-being and physical self-concept. No significant associations were observed for non-recreational screen time.
Wallenius et al. (2010) [147]	Observational study	*n* = 72 school-aged children (39 boys, 33 girls; aged 10 and 13 years)	Mixed digital media use (mobile phones, gaming, computer and Internet activities)	Children exposed to ≥3 h/day of digital media use showed a significantly reduced cortisol awakening response compared with those reporting <1 h/day or no use, suggesting altered cortisol regulation and increased physiological stress.
Morin-Major et al. (2016) [148]	Cross-sectional study	*n* = 88 adolescents (41 boys, 47 girls); mean age 14.5 ± 1.8 years	Social media use (Facebook)	Facebook use frequency was not associated with cortisol levels or depressive symptoms. However, a larger Facebook network was associated with higher diurnal cortisol concentrations, whereas greater peer interaction on Facebook was associated with lower cortisol levels.
Orben et al. (2022) [153]	Longitudinal study	*n* = 17,409 adolescents (10–21 years, both sexes)	Social media use	Higher social media use predicted lower life satisfaction during specific developmental windows, with age- and sex-specific differences.
Przybylski et al. (2020) [152]	Cross-sectional cohort study	*n* = 50,212 children and adolescents (4–17 years)	TV-based and device-based screen engagement	Moderate screen engagement (1–2 h/day) was associated with slightly higher psychosocial functioning compared with lower or higher levels of use. The relationship between screen time and psychosocial functioning was small and non-linear.
Semar & Bakshi (2022) [39]	Cross-sectional study	*n* = 100 school-aged children (8–10 years; 27 boys, 73 girls)	Overall screen time (smartphones, tablets, laptops, TV, gaming and online activities)	Higher screen time was positively associated with emotional overeating and other adverse eating behaviours, and negatively associated with physical activity, suggesting potential pathways linking screen use with overweight and obesity risk.

## Data Availability

No new data were created or analyzed in this study. Data sharing is not applicable to this article.

## Abbreviations

The following abbreviations are used in this manuscript:

AAP	American Academy of Pediatrics
ABCD	Adolescent Brain Cognitive Development Study
ACTH	Adrenocorticotropic Hormone
ALT	Alanine aminotransferase
AST	Aspartate aminotransferase
BMI	Body Mass Index
CRH	Corticotropin-releasing hormone
EE	Emotional Eating
FOMO	Fear of Missing Out
GOCS	Growth and Obesity Chilean Cohort Study
GI	Glycemic index
GL	Glycemic Load
GUS	Central Statistical Office
HbA1c	Glycated hemoglobin
HBSC	Health Behaviour in School-Aged Children
HDL	High-Density Lipoprotein
HOMA-IR	Homeostasis Model Assessment of Insulin Resistance
HPA	hypothalamic–pituitary–adrenal axis
OMA	Obesity Medicine Association
SM	Social Media
SCN	Suprachiasmatic nucleus
TG	Triglycerides
UNICEF	United Nations International Children’s Emergency Fund
